# Equal in ashes? Exploring socioeconomic inequalities in lifespan based on obituary data in Austria

**DOI:** 10.1016/j.ssmph.2023.101550

**Published:** 2023-10-31

**Authors:** Susanne Mayer, Michael Berger, Moritz Oberndorfer

**Affiliations:** a Department of Health Economics, Center for Public Health, Medical University of Vienna, Vienna, Austria; b Department Health Economics and Health Policy, Institute for Advanced Studies, Vienna, Austria; c Population Research Unit, Faculty of Social Sciences, University of Helsinki, Helsiniki, Finland; d Max Planck, University of Helsinki Center for Social Inequalities in Population Health, University of Helsinki, Helsinki, Finland; e MRC/CSO Social and Public Health Sciences Unit, School of Health and Wellbeing, University of Glasgow, Glasgow, United Kingdom

**Keywords:** Health inequality, Lifespan, Socioeconomic indicators, Obituary, Data, Regression analysis

## Abstract

Understanding the emergence of and changes in socioeconomic inequalities in lifespan requires reliable, longitudinal data. In the absence of administrative data, published obituaries may be one such alternative source. With the validity of drawing relevant data from obituaries not yet established in population health research, this study addresses this gap by estimating socioeconomic inequalities in lifespan in Vorarlberg, Austria. Data for all individuals (n = 1490) with obituaries published (July to December 2022) in a regional newspaper (market share: 56%) were extracted, including different markers of the deceased's socioeconomic status. Linear regression analyses showed that, on average, individuals with medium-sized obituaries lived 6.02 years (95% CI: 4.19, 7.85) and individuals with the largest obituaries 12.04 years (95% CI: 7.04, 17.04) longer than individuals with small obituaries while blue-collar workers lived 10.50 years (95% CI: -14.51, -6.49) shorter than individuals with no occupation (reported). This socioeconomic gradient is in line with findings based on national data sources, and comparisons with official regional data are promising regarding data representativeness and completeness. With obituary size reflecting different costs (€210–€1626) and thus being a novel marker for financial ability, obituaries could also be a useful, innovative data source internationally for historical analyses or “nowcasting” health inequalities.

## Highlights

• Obituaries are an underused data source in population health research.
• Obituaries can reveal historical socioeconomic inequalities in lifespan.
• They contain useful socioeconomic data in the absence of administrative data.
• Using obituary data, we estimated socioeconomic gradients in lifespan for Austria.
• Our findings support the validity of these data for population health research.

## 1 Background

Socioeconomic inequalities in morbidity and mortality are a major challenge for healthcare systems worldwide (van Lenthe & Mackenbach, 2021). For example, Europeans with high levels of formal education can expect to live substantially longer (men: 2.3–8.2 years, women: 0.6–4.5 years) than individuals with low levels of formal education (Mackenbach et al., 2019). These inequalities emerge irrespective of the socioeconomic indicators used (OECD, 2020). In addition to providing information about population health, socioeconomic inequalities in mortality have far-reaching ramifications for societal cohesion in terms of equity and fairness. As such, they are critical for evidence-informed policy-making. To improve our understanding of the conditions that shape health inequalities across time, reliable, longitudinal and local micro data are a prerequisite (Mielck & Wild, 2021).

However, a lack of relevant data often impedes analyses of how health inequalities emerged and changed in earlier centuries (Kang-Auger et al., 2021). Although historians can on occasion unearth data in the form of censuses or written data of church parishes, these are often not very detailed and limited to specific geographic areas or occupations (Antonovsky, 1967). Previous studies have therefore explored cemetery data as an alternative data source in this historical context (Kang-Auger et al., 2021; Smith et al., 1992), although these are often accompanied by limitations in their usability. For instance, tombstones are exposed to weather, which may affect their condition in the long run, and their cost depends on artistic factors that may be hard to quantify in retrospect. In contemporary research, the problem is of different nature: While administrative data arguably provides datasets that are both large and accurate, these data are usually only made available with a considerable time lag. This, in turn, curbs the possibility of researchers to conduct urgent timely analyses of the socioeconomic gradient in mortality, e.g., during the early onset of the Covid-19 pandemic.

Obituaries are a promising alternative, yet underused source to fill the data gap (Wallace et al., 2019). They are routinely written with the support of funeral staff based on a wide variety of “template texts” (Nwoye, 1992) and can be informative, opinionative or a combination of the two (Fernández, 2006). And while newspapers are not necessarily a stable medium over time either (e.g. in terms of price increases), unlike tombstones, they are not subject to environmental and aesthetic factors. In addition to the standard information included on the name and date of birth and death (based on official documents), information provided in obituaries may be seen as a proxy for what next of kin consider vital and thus may be subject to bias (Wallace et al., 2019). At the same time, since obituaries may overemphasise, underemphasise or omit certain details, that are, in turn, likely to reflect what society holds important and approximate the “larger social milieu's value system” (Barth et al., 2013; Carmon, 2021; Fowler & Bielsa, 2007), substantiating the assumption that obituaries reflect an individual's socioeconomic standing. Indeed, a plausible explanation for the data presented in obituaries appears to be the “social construction and reconstruction of a life and the events” thought to be most important, worth remembering or to be publicly shared (Marks & Piggee, 1999).

While obituaries have previously served as a basis for linguistic and visual analyses (Barth et al., 2013; Carmon, 2021; Ergin, 2010, 2022; Fowler & Bielsa, 2007; Hamann, 2016a, 2016b; Hess & Pinto, 2020; Heynderickx & Dieltjens, 2016; Kelly et al., 2019; Nwoye, 1992), studies in health sciences are scarce. Resting on the assumption that the activities stated in an obituary may have affected the deceased's health by forming their experiences (Wallace et al., 2019), earlier research was mostly limited to assessing the lifespans of individuals with specific occupations (Allison & Leger, 1999; Gomez, 2017; Vieweg et al., 2003; Wright & Roberts, 1996). Recently, one study looked into the effect of religious affiliation on longevity by drawing on obituary data (Wallace et al., 2019). In another recent study, obituaries were used to extract data for epidemic surveillance in the early stages of the COVID-19 pandemic (Buonanno & Puca, 2021). In terms of the generalisability of obituary data, early UK research investigating the longevity of Methodist ministers concluded that such data may be biased if present and generally seem incomplete and unrepresentative (Allison & Leger, 1999). At the same time, a US study that used obituaries for a content analysis and compared the data with official death statistics yielded promising results in terms of representativeness regarding age and gender (Marks & Piggee, 1999). For historical analyses of the earlier 20th century and before, especially in the absence of administrative data, obituaries could therefore be a potential alternative data source to study health inequalities.

Against this background, this research sets out to conduct a proof-of-concept study by estimating the current socioeconomic inequalities in lifespan based on obituary data from 2022. Our main intention with this paper is to demonstrate and empirically validate the feasibility and soundness of this methodological approach and thereby widen the possibilities for historical and contemporary research on health inequalities in the absence of administrative data. The comparison of the estimated socioeconomic gradient in lifespan with similar studies further allows to somewhat gauge any potential bias in estimates derived from obituaries. The empirical evidence comes from the Austrian federal state of Vorarlberg, which is of interest due to the strong regional tradition of publicly announcing deaths in newspaper obituaries. The second addition of this article to the literature is that it is the first study in population health research assessing the socioeconomic inequalities in the lifespan of a general population drawing on the underused data source of obituaries. Our results substantiate our premise that obituary data offer an adequate reflection of the socioeconomic gradient in mortality albeit with the caveat that higher socioeconomic groups may be slightly overrepresented as indicated by a higher mean age at death in the obituary sample compared to data from death registries. Nevertheless, we find that the estimated socioeconomic gradient is roughly in line with comparable studies, which bolsters the case of using obituary data research on health inequalities.

## 2 Material and methods

### 2.1 Study design

The study is based on a complete survey of obituaries published in the regional newspaper *Vorarlberger Nachrichten* (VN) between July 1, 2022 and December 31, 2022. Re-established after World War II in 1945, the VN is a daily newspaper with the highest regional market share (56% in 2017; Vorarlberger Nachrichten (2021)) in Vorarlberg (406,886 inhabitants; 2022). All of the obituaries are published in a specific section of the print version of the newspaper. Together with the deceased's name, date of birth, date of death, photo and a grief quote, these obituaries typically include details on the time and place of the funeral and the location and names of the bereaved as standard (minimum) information (Vorarlberger Nachrichten, 2022, Vorarlberger Nachrichten, 2022). Official documents including the deceased's birth certificate, proof of citizenship, marriage certificate and divorce decree, as appropriate, need to be provided when placing an obituary, thus ensuring the accuracy of such information (Vorarlberger Nachrichten, 2022, Vorarlberger Nachrichten, 2022). For this study, relevant data were extracted manually (January 2023; by one author) from the newspaper's freely accessible website (Vorarlberger Nachrichten, 2022, Vorarlberger Nachrichten, 2022), which includes all obituaries from the print version. Independent checks of all extracted data were conducted and any discrepancies resolved via discussion.

Data extracted from the obituaries include the name of the deceased as a unique identifier together with the date of birth and date of death. In addition, the deceased's sex and the number of published obituaries were recorded. Finally, several potential markers of the deceased's socioeconomic status were extracted as variables of interest. Firstly, the size of the obituary, i.e. the space in mm^2^ it takes up in the newspaper was recorded. In the case of multiple obituaries, the largest obituary size was used in the analysis. As presented in Table 1, the variable was grouped into small, medium and large obituaries based on the five standard obituary size options and corresponding to €210, €499 and €1234–€1626 in publication costs according to the VN's price list (Vorarlberger Nachrichten, 2022, Vorarlberger Nachrichten, 2022). Previous research highlighted that the social esteem of a deceased person may be manifested in a larger obituary size (Barth et al., 2013; Eid, 2002). The cost of publishing the obituary is covered by the ordering party, which, in Vorarlberg, is surviving family members in almost all cases but may include friends, (former) employers and/or institutions with social/honorary membership including fraternities, particularly in the form of additional obituaries. If available, the family may cover the costs of placing the obituary from the deceased's estate. Therefore, the size of the obituary, especially when placed (and presumably paid for) by the family as next of kin, may be considered a reflection of the ordering party's and the deceased's financial ability (Barth et al., 2013; Ergin, 2010).

**Table 1:** Prices for placing an obituary in the *Vorarlberger Nachrichten* (print)^a^ by size (2022).

Variable	Size (in mm, mm^2^)	Price in EUR
Small	138 (length), 810 (width), 11,178 (area)	210
Medium	138 (length), 167 (width), 23,046 (area)	499
Medium	278 (length), 81 (width), 22,518 (area)	499
Large	138 (length), 253 (width), 34,914 (area)	1234
Large	27.8 (length), 167 (width), 46,426 (area)	1626

a Note: All prices include a colour photo and additional publication of the obituary on the newspaper's website (Vorarlberger Nachrichten, 2022, Vorarlberger Nachrichten, 2022), where fictional examples of the different obituary sizes (with identical content) are also available (Vorarlberger Nachrichten, 2022, Vorarlberger Nachrichten, 2022).

Secondly, the academic grades used as titles in obituaries were extracted as an indicator of the deceased's educational attainment. Based on the International Standard Classification of Education (ISCED), we used this information as a binary indicator variable for tertiary education, including ISCED levels 6 (e.g. bachelor degree), 7 (e.g. master degree) and 8 (e.g. doctoral degree) (ISCED, 2023) versus ISCED levels <6.

Thirdly, the deceased's (former) occupation was recorded to capture their professional career if listed in the obituary. Adopting the Erikson-Goldthorpe-Portocarero scheme (Dhungel et al., 2021), we differentiated between blue-collar and white-collar workers for those individuals with occupation-related information given in the obituary and no occupation/no occupation reported for the remaining individuals. Note that this distinction limits work to the formal market and excludes informal labour, for example at home as a caregiver or housewife. According to earlier research, stating the deceased's occupation in obituaries is an important way of assigning status both to the deceased and those associated with the person (Ergin, 2010).

Fourthly, having provided voluntary services and/or belonging to a social club and/or being in receipt of a badge of honour for special achievements or longstanding membership (referred to as ‘voluntary service’ in tables and figures) was included as another variable of interest. Although not necessarily a socioeconomic indicator, voluntary service or honorary membership could reflect higher social integration and connectedness (Ergin, 2010), which has previously been found to be associated with longevity (Darin-Mattsson et al., 2017).

Fifthly, having a call for donations to charitable or cultural causes included in the obituary – as opposed to a call for flower donations or no such call for donations listed at all – was assumed to be as a proxy for higher social standing (Ergin, 2010). Being in a position to assist others may be both an indicator of philanthropy (Ergin, 2010) and of the economic capital of the deceased and their family.

Finally, reflecting the title of this study (“Equal in ashes?”), mention of urn burial in the obituary was additionally extracted for descriptive purposes.

### 2.2 Data analysis

Based on the date of birth and date of death, we calculated the lifespan in years to two decimal points (and for comparison the exact age at death, i.e. an individual's lifespan rounded down); with the exception of obituary size and the type of occupation included as categorical variables, all other variables were binary. Data analysis was limited to individuals for whom the date of birth or, as an alternative minimum, age at death was reported. Associations between lifespan and each socioeconomic indicator were estimated by separate multivariable ordinary least square regression models with robust standard errors, for all individuals (with sex as a control variable) and men and females separately.

**Fig. 1:**
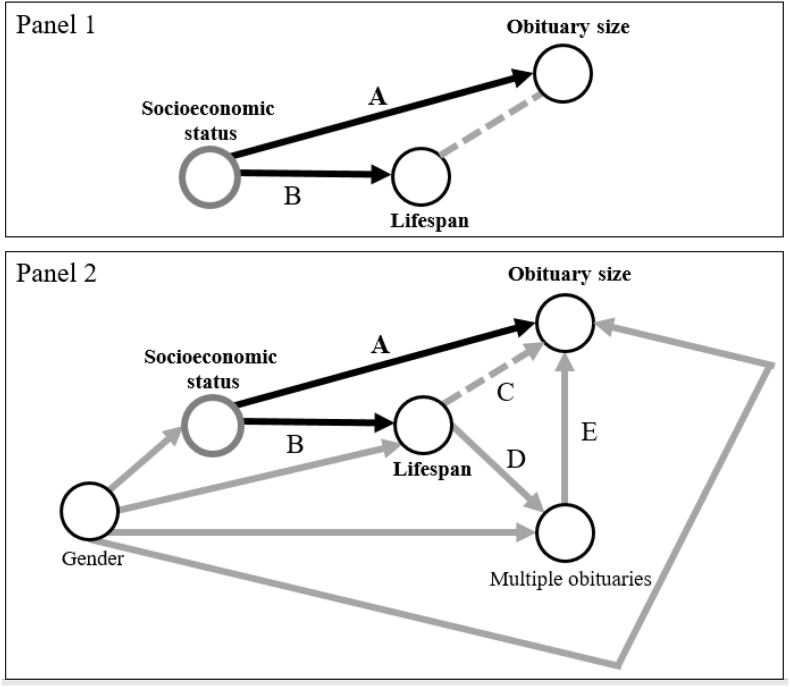
Adapted directed acyclic graph (DAG) illustrating the relationship between socioeconomic status, lifespan and obituary size among deceased Note: Panel 1 depicts the non-causal association (indicated by a dashed line) between obituary size and lifespan that we exploit to estimate the association between (unobserved) socioeconomic status (SES) and lifespan: as SES is associated with lifespan of the deceased and assumed to be associated with obituary size, obituary size and lifespan become associated through their respective connection with SES even if obituary size and lifespan share no causal relationship. The association between obituary size and lifespan is therefore an approximation of the association between the unobserved SES (indicated by grey circle) and lifespan of the deceased. The letters A and B refer to explanations of the assumed relationships provided in the Methods section. In Panel 2, we introduce additional considerations relevant to estimate the targeted association between obituary size and lifespan. The letters A, B, C, and D refer to our detailed explanations of the assumed relationships provided in the Methods section.

It is important to recall that we are not aiming to estimate a causal effect of socioeconomic status on lifespan in this paper. Instead, while drawing on multiple socioeconomic markers, we are also exploring the plausible association between a deceased's true socioeconomic status during their lifetime and the size of their obituary, as illustrated in Fig. 1. We use the association between socioeconomic status and obituary size (A) to estimate the association between socioeconomic status and lifespan (B) when better information is not (yet) available (panel 1 in Fig. 1). However, this analytical approach can be flawed if lifespan has an effect on the obituary size (panel 2 in Fig. 1). For example, when individuals die unexpectedly at a young age and their community might conceive the death as more tragic and therefore show stronger bereavement. In such cases, age at death could directly influence obituary size (C). As another example, dying during or shortly after employment may lead to multiple obituaries as employers might publish obituaries for their former employees (D). Because employers usually have more financial resources than the families of the deceased, (additional) obituaries published by employers might be larger (E). In this case, shorter lifespan may be associated with separate and more expensive obituaries. In Vorarlberg, multiple obituaries seem more common among younger individuals. To mitigate these problems and accommodate for the regional context, we collected data on the number of obituaries by person and used this variable as covariate in our models.

Data extractions were conducted in Microsoft Excel (2016) and all statistical analyses in Stata 17. Statistical significance was assumed at p ≤ .05. The Medical University of Vienna's ethics committee confirmed that ethics approval was not required for this study. The minimum dataset together with the codes for the presented analyses are openly available (Mayer et al., 2023).

## 3 Results

After excluding six individuals (seven obituaries) with missing data on lifespan, the analyses covered 1484 deceased persons. Table 2 gives descriptive information on the study sample. Mean lifespan was 80.99 years (based on exact age at death: 80.48 years), 78.40 years for men and 83.70 years for women, with the latter representing 48.79% of the sample. Medium-sized obituaries were most common (81.54%). For 3.10% of the deceased, tertiary education was reported, and for 86.12% no profession was mentioned. Information on volunteering and/or honorary memberships was given for 9.10% and a call for charitable donations was included for 22.91%. The majority of the deceased (56.81%) were cremated.

**Table 2:** Demographic and socioeconomic characteristics of deceased individuals with obituaries published in Vorarlberg (1 July to December 31, 2022).

	Total sample (n = 1484)		Men (n = 760)		Women (n = 724)	
	N	**Mean (SD) or %**	N	**Mean (SD) or %**	N	**Mean (SD) or %**
**Mean age at death**	1484	80.99 (12.97)	760	78.40 (13.24)	724	83.70 (12.11)
**Obituary size**						
Small	244	16.44	130	17.11	114	15.75
Medium	1201	81.54	610	80.26	600	82.87
Large	30	2.02	20	2.64	10	1.38
**Multiple obituaries**	254	17.12	200	26.32	54	7.46
**Tertiary education**	46	3.10	44	5.79	2	0.28
**Profession**						
None (reported)	1278	86.12	594	78.16	684	94.48
Blue collar	60	4.04	51	6.71	9	1.24
White collar	146	9.84	115	15.13	31	4.28
**Voluntary service**	135	9.10	116	15.26	19	2.62
**Call for donations**	340	22.91	171	22.50	169	23.34
**Urn burial**	843	56.81	425	55.92	418	57.73

**Fig. 2:**
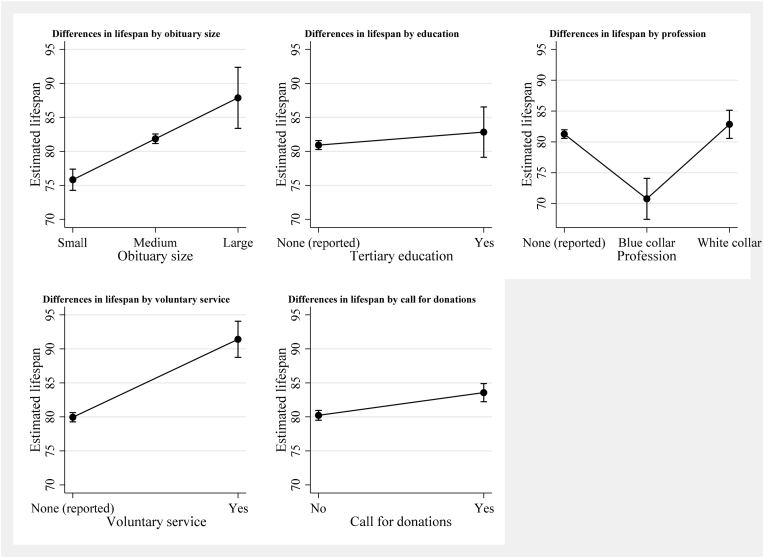
Mean differences in lifespan by collected markers based on obituaries published in Vorarlberg (1 July to December 31, 2022; n = 1484) Note: Regression analyses were run separately for each variable of interest and controlled for sex and multiple obituaries.

In the regression analyses, larger obituary sizes were associated with longer lifespans (Table 3, Fig. 2, Appendix A Figures). Individuals with medium-sized obituaries lived 6.02 years longer (95% CI: 4.19, 7.85) than people with small obituaries and individuals with large obituaries lived 12.04 years longer (95% CI: 7.04, 17.04). Despite not assuming a linear relationship between obituary size and lifespan in our regression model, our results still show the linear socioeconomic gradient in lifespan (see Fig. 2). Having a blue-collar worker position mentioned compared to none was related to a shorter lifespan (-10.50 years, 95% CI: -14.51, -6.49) while volunteering and/or honorary memberships (11.45 years, 95% CI: 8.05, 14.86) and calls for charitable donations (3.34 years, 95% CI: 1.82, 4.85) were associated with longer lives. Generally, females lived longer (5.30 years, 95% CI: 4.01, 6.59) than men and individuals with multiple obituaries shorter (-7.19 years, 95% CI: -9.24, 5.14) than individuals with just one obituary (results not shown).

**Table 3:** Association of lifespan with socioeconomic markers based on obituaries published in Vorarlberg (1 July to December 31, 2022).

	Total sample (n = 1484)						Men (n = 760)						Women (n = 724)					
Age at death	Coefficient		Robust SE	95% CI		R^2^	Coefficient		Robust SE	95% CI		R^2^	Coefficient		Robust SE	95% CI		R^2^
**Obituary size**						0.10						0.05						0.09
Small	reference																	
Medium	6.02	*	0.93	4.19	7.85		6.33	*	1.20	3.97	8.69		5.53	*	1.45	2.68	8.38	
Large	12.04	*	2.55	7.04	17.04		10.44	*	3.18	4.19	16.68		15.04	*	3.96	7.27	22.82	
**Tertiary education**	1.90		2.26	-2.54	6.34	0.07	1.87		2.30	-2.64	6.38	0.02	-8.22		8.78	-25.46	9.01	0.06
**Profession**						0.09						0.06						0.07
None (reported)	reference																	
Blue collar	-10.50	*	2.05	-14.51	-6.49		-9.35	*	2.31	-13.88	-4.83		-15.62	*	3.42	-22.33	-8.90	
White collar	1.59		1.37	-1.11	4.28		3.40	*	1.51	0.44	6.36		-4.46		2.95	-10.24	1.33	
**Voluntary service**	11.45	*	1.74	8.05	14.86	0.11	11.01		2.01	7.07	14.94	0.07	10.72	*	3.47	3.91	17.52	0.07
**Call for donations**	3.34	*	0.77	1.82	4.85	0.08	4.64	*	1.11	2.46	6.81	0.04	2.09	*	1.07	0.00	4.18	0.06

Note: Regression analyses were run separately for each variable of interest, with regressions on the total sample controlled for sex and multiple obituaries, and sex-stratified regressions controlled for multiple obituaries only. *p ≤ .05.

The sex-stratified regression analyses indicate similar associations for males and females, with one exception. For men, having a white-collar worker position listed compared to none correlated with a longer life (3.40 years, 95% CI: 0.44, 6.36).

## 4 Discussion

The findings presented on socioeconomic inequalities in lifespan based on obituary data in Vorarlberg are in line with the well-established socioeconomic gradient in mortality in Austria (Doblhammer et al., 2005; Klotz, 2007; Klotz et al., 2019; Mackenbach et al., 2004; Schwarz, 2007; Spitzer et al., 2022). For example, in 2019, men with tertiary education had a longer life expectancy by 6.5 years than men with minimum compulsory schooling while for women this difference amounted to 3.7 years (Statistics Austria, 2021). In general, however, earlier Austrian studies were limited in scope with regards to socioeconomic indicators and only focussed on limited age ranges, population groups and time periods. This study adds to the literature by demonstrating the usefulness of obituaries to study lifespan inequalities in situations where superior (e.g., administrative) data sources are not (yet) available. Next to socioeconomic markers, the screened obituaries also held data on the deceased's social network, end-of-life arrangements and support, for example. This study set out to be a proof-of-concept for future research using (historical) obituary data. The fact that both obituary and administrative data provide a comparable picture in terms of inequality in mortality lends support to use the former as a data source in population health research, especially in studies for which alternative data may be absent. Such research may also provide impetus and input for future theory building on, for example, the historical development of socioeconomic inequalities.

Obituary size correlated strongly with lifespan. According to the descriptive analysis presented here, affording the largest obituary sizes compared to smaller ones was related to men living almost eleven years longer and women 15 years longer. Funeral costs including obituaries may be covered by the deceased's estate. As the obituary size and its cost also mirrors the deceased's family's ability to pay for the public announcement of a death, the size may indeed reflect financial resources (Barth et al., 2013) and could even be considered as ‘post mortem’ conspicuous consumption. Our findings are also in line with a historical analysis of lifespan and its association with tombstone size in tertiles of height as a proxy for socioeconomic status in Canada (Kang-Auger et al., 2021): Individuals with larger and more expensive tombstones were found to have lived 9.6 years longer than individuals with smaller tombstones.

Our indicator of tertiary education was not associated with a statistically significant mean difference in lifespan, but our study was underpowered to estimate this association due to the small number of obituaries reporting the academic degree of the deceased. Moreover, this finding emphasises the relevance of obituary size, a variable available for all individuals, as a socioeconomic indicator for time periods in which fewer individuals had access to higher education, especially women. Occupational status, another commonly used proxy for an individual's socioeconomic status (Mielck & Wild, 2021), may also be problematic when studying inequalities in past centuries due to lower (especially female) labour force participation. In line with earlier research (Marks & Piggee, 1999), details on any former occupation were not commonly reported in obituaries in our data for either sex, potentially mirroring the lacking societal recognition for the majority of common occupations. In addition, female labour force participation for the main cohort included in our study was low in Austria (Butschek, 1974). In our study, both males and females with a listed blue-collar position had a shorter lifespan than those with no occupation (reported) in their obituary. However, with data on a blue-collar profession only being reported for nine women, this finding should be interpreted with caution. For men, having a white-collar position listed compared to none was associated with a longer life.

In contrast to administrative and cemetery data, obituaries also hold additional information on the deceased's social connectedness. For example, having provided voluntary services or being awarded honorary membership was associated with a longer life in Vorarlberg, which is line with international literature (Wallace et al., 2019). Although not a classic socioeconomic proxy, it may be hypothesised that being healthy enough to engage in volunteering, especially in the late period of life, is related to a higher socioeconomic status. Equally, such service provision is argued to have salubrious effects (Jenkinson et al., 2013). Finally, having a call for charitable donations included in the obituary was related to a longer lifespan. This could mean that the relatives of the deceased do not need to rely on donations to cover the costs associated with the funeral, which could thus represent another potential socioeconomic indicator of the deceased. Further qualitative research may be helpful in gaining a better sense of the plausibility of these hypotheses.

Women were found to live longer than men by around five years, which is in line with the gender gap in current life expectancy (Bundeskanzleramt, 2021) and age at death (Murtin & Lübker, 2022) in Austria. Having multiple obituaries was found to be related to significantly shorter lifespans. In sensitivity analyses excluding multiple obituaries as a control variable, the overall findings still held true but with slightly smaller differences in lifespan by obituary size.

## 5 Limitations

Despite potential advantages over other data sources (Buonanno & Puca, 2021; Wallace et al., 2019), using obituary data for health inequalities research may have some limitations, specifically related to selection bias, missing data, reverse causation, and unobserved confounders. Firstly, certain population subgroups may not be fully represented (Buonanno & Puca, 2021). For example, financial barriers to placing even the smallest obituary may be a prohibitive factor and newspaper obituaries may not be common among certain religious/ethnic groups. If true, this would mean that the inequalities in lifespan identified by obituary size are potentially an underestimate. Indeed, looking at the names included in our database as a first, albeit imprecise, approach, in the light of the official 28% proportion of people with foreign background in Vorarlberg (Statistik Austria, 2023a), it seems possible that this sub-group is not proportionally covered in obituaries and a selection bias exists. Secondly, obituary size might correlate with the reporting of other variables of interest, i.e. small obituaries not allowing the inclusion of alternative socioeconomic markers for reasons of space. However, examples in our dataset confirm that it is technically possible to fit comprehensive socioeconomic information in small-sized obituaries as well. Along these lines, while the demographic data included are backed up by official documentation, some socioeconomic data may be subject to measurement inaccuracies (e.g. professional activities, voluntary services). However, we agree with other authors that, from a societal viewpoint, the (non-)reporting of data is not coincidental (Barth et al., 2013; Carmon, 2021; Fowler & Bielsa, 2007). Finally, our application of obituary data to estimate inequalities in lifespan is a descriptive and explorative exercise. Although it is not meant to uncover causal effects, such descriptive results still valuably add to the scientific discourse (Fox et al., 2022; Wallace et al., 2019).

## 6 Data completeness and representativeness

Comparisons of our data with regional official data mostly paint a favourable picture in terms of the representativeness, completeness and (where comparisons are possible) accuracy of the obituary data, whereby representativeness of the obituary data is more important than completeness when these data are used for health inequalities research. With 1490 deaths reported in the obituaries published between July and December 2022 and 1813 deaths registered between July and December 2021 as the latest available data (Statistik Austria, 2023b), coverage of deaths in obituaries is presumed to be around 82% and also confirms the strong regional relevance of obituaries as a means of public communication. The sex distribution (51.01% males in obituaries, 51.52% males in death registries in 2021) is nearly identical in both data sources (Statistik Austria, 2023b). At 80.48 years (exact age at death), mean age at death in obituaries is 1.91 years higher than in the regional death registries (78.57 years in 2021) (Statistik Austria, 2023b). In the context of previous studies that showed the positive association of higher socioeconomic status with longevity, this slightly higher estimate of the socioeconomic gradient likely reflects the issue of a selection bias in the sample size: there may be disproportionately fewer obituaries for individuals with lower socioeconomic status. Indeed, comparing the proportion of deceased individuals with tertiary education in the obituary data (3%) and official statistics (4% in 2020; Statistik Austria (2023c)), it seems that academic degrees are either somewhat underreported or individuals with tertiary education are slightly underrepresented in obituaries published in the VN. Despite these limitations, obituary data is a useful data source when more accurate official data are not (yet) available.

## 7 Conclusions

Overall, the comparison of our findings with official statistics on an aggregate level supports our hypothesis that the obituaries capture the deceased's individual socioeconomic status. This study suggests that data collected from obituaries are a potentially valid and rich alternative data source when studying inequalities in lifespan on a regional level. The approach seems most suited for areas where mourning is not exclusively a private experience but also “shared with the public” (Fowlkes, 1990 in Barth et al., 2013), i.e. also outside Vorarlberg under the condition of reasonable data representativeness. Thus, obituary data could be particularly useful when administrative data is unavailable, for example for historical analyses. In Austria, this is the case for the period before 1980 (Antonovsky, 1967; Klotz, 2010). As data from VN obituaries published after 1945 are electronically available and accessible, they could be used to partially fill this gap. However, our findings also provide the important insight that lower socioeconomic groups are potentially underrepresented in obituary data, leading to a slight upward bias in estimates for longevity and underestimation of lifespan inequalities.

Our results suggest that using obituary size/price as a socioeconomic indicator of the deceased may be the most cost-effective approach to studying (historical) inequalities in lifespan with these data. In contrast to other information available from obituaries, their size and price is non-missing for all obituaries. This advantage is especially important for periods in which women had worse access to tertiary education and the labour market, making socioeconomic indicators like formal education and occupational status less useful for health inequalities research. While manual data extraction from obituaries is resource intensive, web scraping or implementing legislation to support routine electronic storage has the potential to drastically shorten the time needed for data collection. Apart from allowing historical analyses of health inequalities, these data could provide valuable “nowcasting” of daily (socioeconomic) mortality patterns during natural disasters, pandemics, or other health emergencies.

## Funding

This study did not receive any specific grant from any funding agencies. However, MO was supported by the 10.13039/501100000781European Research Council under the European Union's Horizon 2020 research and innovation programme (grant agreement No 101019329), and grants to the Max Planck – University of Helsinki Center from the 10.13039/501100004012Jane and Aatos Erkko Foundation, the 10.13039/501100004189Max Planck Society, 10.13039/100007797University of Helsinki, and Cities of Helsinki, Vantaa and Espoo. The study does not necessarily reflect the Commission's views and in no way anticipates the Commission's future policy in this area. MO's work was also supported by the 10.13039/501100000265Medical Research Council (MC_UU_00022/2) and the Scottish Government Chief Scientist Office (SPHSU17). The funders had no role in the study design, data collection and analysis, decision to publish, or preparation of the manuscript.

## Author contributions

Conceptualization: SM, MB, MO; Data curation: SM; Formal analysis: SM, MO; Funding acquisition: -; Investigation: SM; Methodology: SM, MB, MO; Project administration: SM; Resources: -; Software: -; Supervision: SM; Validation: SM, MB, MO; Visualization: SM, MO; Roles/Writing - original draft: SM; and Writing - review & editing: SM, MB, MO.

## Ethics approval

The Medical University of Vienna's ethics committee confirmed that ethics approval was not required for this study.

## Declaration of competing interest

The authors declare that they have no known competing financial interests or personal relationships that could have appeared to influence the work reported in this paper.

## Data Availability

Anonymized data has been made available, see details in the methods section.
