# Supplementary material for: Equal in ashes? Exploring socioeconomic inequalities in lifespan based on obituary data in Austria
Source: SSM Popul Health. 2023 Oct 31;24:101550. doi: 10.1016/j.ssmph.2023.101550 (PMC10665934; doi:10.1016/j.ssmph.2023.101550)
Supplement: Multimedia component 1 [file mmc1.docx]

Equal in ashes? Exploring socioeconomic inequalities in lifespan based on obituary data in Austria

**Appendix A**

**Figure 1: Mean differences in lifespan by socioeconomic markers based on obituaries published in Vorarlberg (n=1484)**

Note: Regression analyses were run separately for each variable of interest and controlled for sex and multiple obituaries.

**Figure 2: Mean differences in lifespan by socioeconomic markers for men based on obituaries published in Vorarlberg (n=760)**

Note: Regression analyses were run separately for each variable of interest and controlled for multiple obituaries.

**Figure 3: Mean differences in lifespan by socioeconomic markers for women based on obituaries published in Vorarlberg (n=724)**

Note: Regression analyses were run separately for each variable of interest and controlled for multiple obituaries.
